# Human Reproduction and Disturbed Genomic Imprinting

**DOI:** 10.3390/genes15020163

**Published:** 2024-01-26

**Authors:** Thomas Eggermann

**Affiliations:** Institute for Human Genetics and Genomic Medicine, Medical Faculty, RWTH University Aachen, Pauwelsstr. 3, D-52074 Aachen, Germany; teggermann@ukaachen.de; Tel.: +49-241-8037285; Fax: +49-8082394

**Keywords:** genomic imprinting, reproductive failure, placenta, pregnancy issues

## Abstract

Genomic imprinting is a specific mode of gene regulation which particularly accounts for the factors involved in development. Its disturbance affects the fetus, the course of pregnancy and even the health of the mother. In children, aberrant imprinting signatures are associated with imprinting disorders (ImpDis). These alterations also affect the function of the placenta, which has consequences for the course of the pregnancy. The molecular causes of ImpDis comprise changes at the DNA level and methylation disturbances (imprinting defects/ImpDefs), and there is an increasing number of reports of both pathogenic fetal and maternal DNA variants causing ImpDefs. These ImpDefs can be inherited, but prediction of the pregnancy complications caused is difficult, as they can cause miscarriages, aneuploidies, health issues for the mother and ImpDis in the child. Due to the complexity of imprinting regulation, each pregnancy or patient with suspected altered genomic imprinting requires a specific workup to identify the precise molecular cause and also careful clinical documentation. This review will cover the current knowledge on the molecular causes of aberrant imprinting signatures and illustrate the need to identify this basis as the prerequisite for personalized genetic and reproductive counselling of families.

## 1. Introduction

**Figure 1:**
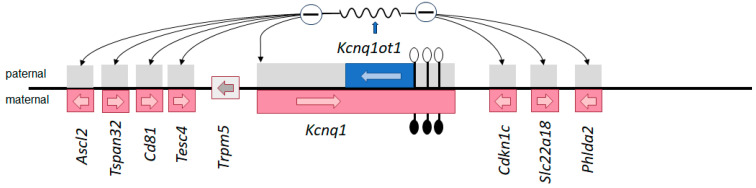
The imprinted *Kcnq1* gene cluster in the mouse embryo (not all genes are shown) as an example of the complex tissue- and time-specific regulation of genomic imprinting. The DMR is within the promoter of the non-coding RNA Kcnq1ot1: on the maternal allele (genes transcribed from the maternal allele are shown in red), the DMR is methylated (filled lollipops), whereas the paternal allele is unmethylated (empty lollipops) and transcribed (blue). The *Kcnq1ot1* transcript functions bidirectionally throughout the locus and silences several cis genes (gray). In mouse embryos, there are two types of placenta-specific imprinted genes in the region, those which are silenced on the paternal allele only in placental tissues (*Ascl2, Tspan32, Cd81, Tssc4*) and those with silencing in both placental and embryonic tissues (*Kcnq1, Cdkn1c, Slc22A18, Phlda2*). The region is syntenic with the human imprinting center 2 (IC2) in 11p15 associated with BWS; it should be noted that pathogenic variants of the maternally expressed *CDKN1C* allele can be identified in BWS and SRS.

The term genomic imprinting describes the epigenetic phenomenon of the expression of certain genes being monoallelic and parental-origin-specific. Accordingly, either the maternal or paternal allele is active, whereas the other gene copy is silenced (Figure 1). Genomic imprinting predominantly occurs in placental mammals, and it has been suggested that its evolution is linked to the competition for nutrients between the fetal and maternal metabolism during pregnancy. This conflict hypothesis of genomic imprinting [1] is supported by the observation that paternally expressed imprinted genes often favor larger offspring, whereas maternal ones suppress fetal growth.

**Figure 2:**
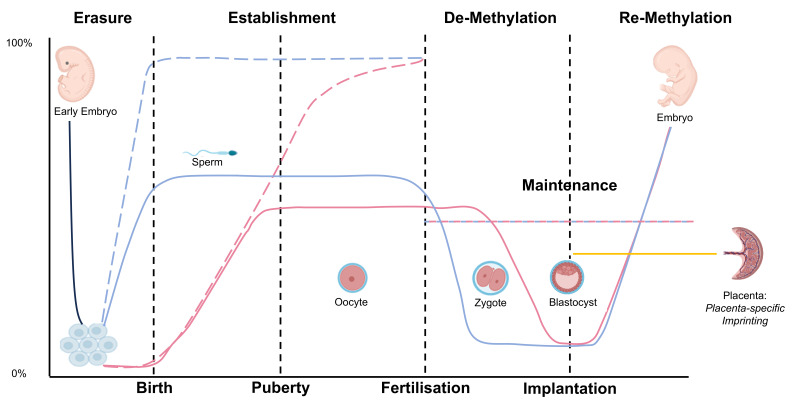
The imprinting cycle of life (based on data from mice, but with similarities in humans). In primordial germ cells (PGCs) in the embryo, DNA methylation is generally erased (solid black line). In the following, de novo methylation signals are established during gametogenesis. Here, a sex-dependent dichotomy can be observed: in the oocytes, general DNA methylation is established after birth (solid red line), whereas in sperm, it occurs soon after demethylation in the embryo and is finished at birth (solid blue line). Establishment of genomically imprinted DMRs can be observed at the same time (dashed lines) but demethylation does not affect imprinted DMRs. Imprinted loci maintain their methylation, whereas non-imprinted loci are rapidly demethylated, either actively in the case of paternal genomes or passively following DNA replication in the case of maternal genomes. It should be noted that the imprinting signature is different for several genes in the placenta and the embryo. (Pictograms have been taken from BioRender.com (accessed on 23 January 2024)).

A key feature of imprinted genes is their organization into clusters, which are regulated by long-range *cis*-acting imprinting control regions (ICs). Molecularly, ICs are characterized by germline differentially methylated regions (gDMRs) (Figure 1). Their methylation status is transmitted via the gametes at fertilization and stably maintained in the somatic cells throughout development (Figure 2). Additional DMRs in the same imprinting cluster—if present—are less stable, and their methylation is established during post-implantation development (somatic DMRs (sDMRs)). They are dependent on hierarchical interactions with neighboring gDMRs.

Molecularly, imprinted genes and their regulation are orchestrated by allelic DNA methylation (i.e., 5-methylcytosine (5mC)), histone modifications and non-coding RNAs [2], with an impact on the spatial chromosomal structure and the accessibility of imprinted gene transcription. In addition, imprinted gene clusters are interconnected [3], and for several imprinted loci, a modulation in expression by *trans*-acting factors has been demonstrated (e.g., [4,5]).

## 2. Disturbances of Genomic Imprinting

Many imprinted genes are involved in human development and growth pathways; therefore, it is not surprising that disturbances of the fine-tuned expression of imprinted genes affect human reproduction, pregnancy and embryogenesis. Accordingly, the majority of the currently known imprinting disorders are associated with aberrant fetal growth, placental abnormalities and pregnancy complications (e.g., pre-eclampsia, preterm birth) (Table 1).

Currently, four different types of molecular alterations affecting ICs and their function have been identified (for review, see [17]):

- Uniparental disomy (UPD), describing the exceptional inheritance of a chromosomal region from the same parent, i.e., either from the mother solely (maternal UPD) or from the father (paternal).
- Pathogenic structural variants (chromosomal SVs, i.e., deletions, duplications).
- Pathogenic single nucleotide variants (SNVs).
  These above three molecular subtypes affect the DNA sequence of imprinted genes directly.
- Imprinting defects (ImpDefs) are characterized by an aberrant methylation signature of gDMRs, without changes in the underlying DNA sequence.

All four molecular subtypes disturb the balanced expression of genes regulated by genomic imprinting, either by silencing the affected allele or activating it. Molecular diagnostic testing should not only aim to identify the disease-causing imprinted locus to confirm a clinical diagnosis but should also determine the molecular subtype. The reason is that for several ImpDis, the precise molecular subtyping is a prerequisite for a personalized clinical management and is also required for reproductive and genetic counselling [18].

### 2.1. UPD/Aneuploidies

Causes: UPD results from chromosomal nondisjunction or rearrangements [19] and therefore represents chromosomal aberrations. It can affect whole chromosomes, but also segmental UPD of only the imprinted region has been described (for review, see [20]). In the case of whole-chromosome UPD, predisposing factors for its formation are increased maternal age in the case of trisomy rescue and (familial) SVs like Robertsonian translocations [18].

Consequences: The consequences of UPD and its mode of formation are the disturbance of genomic imprinting and thereby ImpDis. In addition, UPD harbors the risk of reduction of parental heterozygosity into homozygosity for a pathogenic (autosomal) recessive variant and placental mosaicism for chromosomal trisomy in the case of trisomy rescue, associated with placental malfunction (for review: [21]).

### 2.2. SVs

Causes: Structural chromosomal variants (SVs) cause ImpDis if an imprinted gene region is affected (for review, see [18]). In fact, several imprinted regions, their function and interaction, as well as their associated phenotypes, could be further characterized in patients with SVs in these regions (e.g., TS14 and KOS14: [22]).

Consequences: As described before, SVs may cause predisposition to UPD formation. However, the major consequence of SVs in the case of imbalance is the disturbance of the fine-tuned expression of imprinted genes. The resulting ImpDis phenotype might be additionally modified by the size and gene content of the affected chromosomal region. SVs can be inherited from generation to generation, and depending on the sex of the parent transmitting the variant, a broad range of clinically altered offspring can occur in these families, including miscarriages and molecularly opposite ImpDis like SRS and BWS [23]. Additionally, small CNVs occur within DMRs and other regions relevant to the status of methylation (see Section 6.1).

### 2.3. SNVs

Causes: SNVs in ImpDis can affect both imprinted genes (Table 1) and non-imprinted genes.

Consequences: In both scenarios, they can occur de novo or be inherited. In the case of SNVs in the coding regions of imprinted genes (including intronic sequences relevant to proper splicing), they only become clinically relevant if they affect the expressed parental allele. Examples are pathogenic SNVs in *IGF2,* which are associated with SRS only if they are inherited from the father (for review, see [24]). Accordingly, they can be transmitted over generations without clinical consequences if the sex of the transmitting family members is the same and is associated with the silencing of the imprinted gene [17]. Finally, if SNVs are localized in imprinted gene regions relevant to the regulation of its imprinting status, they might affect the methylation status (see Section 6.1).

Non-imprinted genes causing ImpDis are members of the methylation machinery and therefore do not directly affect the phenotype, but they cause ImpDef (see ImpDefs) phenotypes if they belong to the respective physiological pathways, like *PLAG1* and *HMGA2,* which have been suggested to be functionally linked to IGF2 in SRS [25].

As pathogenic variants in imprinted genes are inherited autosomal-dominantly with incomplete penetrance due to the sex of the contributing parent, they represent a challenge in genetic counselling. In some ImpDis, (epi)genotype–phenotype correlations can be observed, and this also accounts for SNVs. An example is *CDKN1C* in 11p15.5, as BWS patients with pathogenic SNVs in this gene show some clinical peculiarities [26]. *CDKN1C* can cause both BWS due to loss-of-function variants or SRS due to gain-of-function variants [27].

It should be noted that in some ImpDis, pathogenic SNVs are currently the only known disease-causing types of molecular disturbances (for review, see [17]).

### 2.4. Imprinting Defects (ImpDefs, Epimutations)

Causes: The molecular cause of the majority of identified ImpDefs is currently unknown (primary ImpDefs; see [28]), but there are a growing number of reports of so-called secondary ImpDefs (also called secondary epimutations; see Section 6.1 and Section 6.2). The latter are either the consequence of *cis*-acting DNA variants localized close to the aberrant DMR (e.g., [29]) or *trans*-acting factors (e.g., *ZFP57* [30]) in the fetal genome. *Trans*-acting variants can also be localized in so-called maternal effect genes, i.e., genes which mediate the proper establishment and maintenance of the imprinting signature in the oocyte and in early embryogenesis [31]. In these families, the mother is the carrier of the pathogenic variant, and this variant clinically affects her offspring (see below). However, it is conceivable that a considerable number of ImpDefs will remain classified as primary ImpDefs, as they might be caused by exogenic factors like nutritional status and exposure to chemical pollution [32].

Consequences: The currently known ImpDis in which ImpDefs can be observed are associated with either the hypomethylation or hypermethylation of specific disease-linked DMRs (Table 1). For these entities, different functional consequences have been postulated to cause specific phenotypes, directly by disturbing physiological networks like the IGF2 growth axis in Silver–Russell syndrome [25] or indirectly by affecting imprinting gene networks [4]. In the case of secondary ImpDefs, the underlying genetic variant can affect specific DMRs, like in the case of pathogenic variants in *ZFP57* or in the IC2 region in 11p15.5 [29,33].

However, they can also have an impact on the methylation of several DMRs, an observation called Multi-locus Imprinting Disturbances (MLID; see below). In any case, the patient will show clinical features of ImpDis, either specific to a certain ImpDis like SRS or BWS [34] or an altered or mixed phenotype [35]. As, in some patients, MLID are the result of pathogenic maternal effect variants [36], these families have an increased risk of reproduction failure (see below) [37].

## 3. Genomic Imprinting during Gamete Maturation and Embryogenesis

The regulation of genomic imprinting underlies a so-called life cycle of genomic imprinting and comprises the reprogramming of methylation marks (Figure 2). It starts with a near-complete erasure of DNA methylation in the primordial germ cells (PGCs); subsequently, methylation is re-established in the germ cells. This re-establishment occurs in an asymmetrical fashion, i.e., in male gametogenesis, the new methylation of DMRs starts immediately after demethylation and is completed at birth. In contrast, the gain of methylation in PGCs in females starts after birth and lasts until puberty and adulthood, due to the arrest of the oocytes in the meiosis I prophase. After fertilization, both parental genomes undergo a genome-wide demethylation wave, with the exception of the imprinted DMRs, which remain methylated (for review, see [38]).

The establishment of methylation during gametogenesis is mediated by a complex machinery of numerous factors (for review, see [39]). Both in female and male gametogenesis, Dnmt3a is an essential de novo DNA methyltransferase [40], and its catalytic activity is enhanced by the co-factor Dnmt3L [41]. At the histone level, genomic imprinting is mediated by histone modification: examples are the histone demethylase KDM1B and the histone methyltransferase SETD2, which contribute to the establishment and maintenance of the maternal genomic imprint [42,43]. In addition to this general de novo methylation mechanism, further factors ensure the correct methylation of specific DMRs, such as BORIS and Prmt7, which play a role in the imprinting of the *H19* DMR [44].

After fertilization and during early embryogenesis, imprinted loci are protected from global demethylation, and their methylation marks are maintained. This protection is orchestrated by several methylation-sensitive transcription factors, including ZFP57 and ZNF445 [45,46], and the oocyte-derived subcortical maternal complex (SCMC) [31]. In humans, the relevant role of these factors for the maintenance of the imprinting signature in maturing oocytes and early embryos has been identified according to the identification of pathogenic variants in their respective genes [47].

## 4. Imprinted Genes Involved in Pregnancy

The critical role of imprinted genes in embryogenesis and pregnancy, as well as in later life, has become evident from the identification of ImpDis and their underlying disturbances of balanced genomic imprinting. Imprinted genes control the fetal nutrient supply from the maternal metabolism via the placenta but also regulate fetal growth and fetal demand for maternal resources [48]. After birth, they contribute to the development of metabolic organs and modulate metabolic pathways. Accordingly, major clinical features of disturbed imprinting comprise intrauterine and postnatal growth disturbances, as well as metabolic alterations.

By considering these roles, and based on studies in mice, Charalambous and coworkers [49] suggested three different functional classes of imprinted genes (Table 2). (i) genes with functions restricted to the placenta, (ii) genes that act in the placenta and embryo and (iii) genes with functions after birth.

However, this classification only roughly describes the complex interaction between imprinted genes and their transcription products, and several genes play a role at different stages of ontogenesis.

### 4.1. Imprinted Genes with an Exclusive Role in Placental Physiology

As the placenta is the central organ for the allocation of maternal resources between the fetus and mother at the beginning of life, it is not surprising that its functions require a unique set of factors balancing the need of the fetal and maternal metabolisms. This unique role of the placenta is regulated significantly by imprinted genes, which ensure both cellular differentiation in the placenta as the basis for its function and the transport of nutrients. In fact, several imprinted genes with a sole function in this complex maternal–fetal exchange have been identified (Table 2). Examples are *Mash2* (in humans, *ASCL2)* and *Peg10* which regulate the differentiation of spongiotrophoblast cells into trophoblast giant cells [50,51]. In humans, disturbances of PEG10 and *ASCL2* have not yet been directly linked to ImpDis, but they are affected by the molecular alterations observed in SRS and BWS (upd(7)mat and IC2 LOM, respectively). Thus, placental malfunction in these disorders [8,52] might be attributed to alterations in these genes.

In addition to their impact on cellular differentiation in the placenta, their function might also be disturbed by altered growth and nutrient transport systems [49]. IGF2 reflects the complexity of the spatial and temporal expression of (imprinted) genes, resulting in different transcripts with specific functions in embryogenesis. In the placenta, an *IGF2* transcript derived from the placenta-specific *IGF2* P0 promoter influences the placental size and composition. A lack of this transcript causes reduced passive diffusion and thereby fetal growth retardation, whereas the circulating fetal IGF2 is normal [53].

### 4.2. Imprinting Genes with a Role in the Placenta and the Fetus

Numerous imprinted genes are expressed and probably function in both extra-embryonic and embryonic tissues, and they show a decrease in expression after birth. Though an impact primarily on embryonic growth and development can be expected if they are disturbed, alterations in these genes are also associated with clinical features in later life. These lifelong consequences might be explained according to fetal programming, a hypothesis that states that insults sustained during pregnancy have permanent effects on adult health [54]. In addition to their expression in the placenta, these genes show a broad pattern of expression in different tissues (e.g., (neuro)endocrine tissue, mesodermal derivatives, liver, specific brain tissues). Accordingly, the clinical spectrum of ImpDis comprises growth and metabolic disturbances, hypotonia, dysmorphisms and developmental delay, depending on the DMR specifically altered in the disease.

### 4.3. Imprinted Genes with a Postnatal Role

A third group of imprinted genes with no effect on fetal growth are factors also influencing growth and metabolism; in particular, they contribute to survival in the neonatal period (e.g., *Gnas, Gnasxl, Atp10c*) [49]. As this period is essential for metabolic programming, their disturbance also results in lifelong health issues.

## 5. Fetal Genomic Variants in Imprinted Genes and Their Physiological Impact on Prenatal Growth and Development

As described before, all four currently known molecular alterations known in ImpDis (UPD, SVs, SNVs, ImpDefs) affect the balanced expression of imprinted genes regulated by their specific gDMRs (Table 1) and thereby have an impact on phenotype. However, the pathomechanisms causing the clinical features of ImpDis are widely unknown, and even for those imprinted genes with proven pathogenic variants, their physiological consequences are not fully understood. Examples are two genes in 11p15.5 with an obvious role in growth and development: *IGF2* and *CDKN1C* (Table 2).

The *IGF2* gene is under the control of the imprinting center 1 region (IC1, H19/IGF2:IG-DMR) and is expressed from the paternal methylated allele. Its gene product IGF-II is regarded as a key factor for placental and embryonic growth, but the identification of pathogenic variants in patients with intrauterine and postnatal growth restriction [24] and its postulated contribution to overgrowth in BWS [55] have confirmed the pleiotropic, but currently mainly unknown, effects of this central factor.

A comparable broad impact on growth has been delineated for *CDKN1C,* which is expressed from the maternal allele (Figure 1) and regulated by the maternally methylated IC2 (*KCNQ1OT1*:TSS-DMR). Pathogenic loss-of-function variants in this mitosis inhibitor are commonly known in BWS patients and are localized all over the gene, but, recently, gain of function in the PCNA domain of the gene has been identified in patients with SRS features [27,56]. Though the functional consequences of *CDKN1C* variants are not yet known, *CDKN1C*’s central role in the etiology of 11p15.5-associated disorders has not only become obvious in families with pathogenic variants within the gene but also in patients with structural rearrangements within the IC2 region (e.g., [57]). These familial structural variants have confirmed the negative regulation of *CDKN1C* expression by the non-coding RNA *KCNQ1OT1*. Clinically, pathogenic variants of *CDKN1C* are not only associated with a (prenatal) manifestation of BWS features but also with an increased risk of preeclampsia during pregnancy [52].

## 6. Factors Causing Secondary ImpDefs and Their Role in Reproduction

The establishment and maintenance of imprinting signatures are orchestrated by a complex machinery during embryogenesis, which is prone to multiple disturbances, influenced by genetic and environmental factors. It is therefore not surprising that the number of reports on monogenetic causes of ImpDefs is constantly growing and serves as a basis for family counselling but also for our understanding of the regulation of genomic imprinting. In general, these genetic alterations can be localized spatially close to the DMR, which exhibits aberrant methylation, or they affect members of the machinery in the imprinting life cycle.

### 6.1. Cis-Acting Variants Causing ImpDefs

*Cis*-acting variants causing ImpDefs have meanwhile been reported for several DMRs, and different mechanistic consequences have been suggested.

For several DMRs, SVs and SNVs affecting regulatory elements have been identified, and an effect on the chromatin organization has been suggested. Examples are SNVs and small SVs in the OCT4/SOX2-binding site variants in BWS patients, causing a gain of methylation at the *H19/IGF2*:IG-DMR in 11p15.5 [58], and *STX16* variants on the maternal allele, affecting the *GNAS A/B*:TSS-DMR in 20q13 [59], e.g., via long-range interaction [60].

Another mechanism comprises altered transcripts of genes in imprinted regions with an impact not only on the encoded protein but also on the methylation status of the respective DMR. This mechanism has been identified for the IC2 region in 11p15.5, where *KCNQ1* variants cause haploinsufficiency and thereby LongQT syndrome as well as IC2 loss of methylation [29].

### 6.2. Trans-Acting Variants Causing ImpDefs

By definition, *trans*-acting factors are localized outside the DMR on which they might have an impact, and these factors can be expressed both by the fetal and maternal genomes. In contrast to *cis*-localized factors with an effect on the neighboring DMR, the currently known *trans*-acting factors often cause MLID [4,61] and particularly the loss of methylation of several loci.

An example of a *trans*-acting factor with an impact on the human imprinting expressed by the fetal genome is *ZFP57*, which specifically binds the methylated allele at a subset of gDMRs and thereby contributes to the maintenance of methylation of these loci [62]. Accordingly, pathogenic variants in *ZFP57* result in general hypomethylation [30].

So-called maternal effect variants are an exceptional group of alterations, as the carrier of these alterations are the mothers but they have a relevant impact on pregnancy and offspring. These variants affect proteins like NLRP2, NLRP5, NLRP7 and PADI6 [47], which moderate the proper methylation in the oocytes and early embryos before the fetal genome becomes activated (for review, see [31]). These factors contribute to the subcortical maternal complex (SCMC), a multiprotein structure in the oocyte with multiple functions in zygote progression, cell division and the imprinting cycle of life. Accordingly, pathogenic maternal effect variants are associated with a broad range of reproductive health issues, affecting both the mother and her offspring. With the increasing application of high-throughput next-generation sequencing assays and awareness of this type of non-Mendelian inheritance, there is an increase in reports of maternal effect variants (for review: [33]) and their clinical consequences, including preeclampsia, miscarriages, hydatidiform moles, aneuploidies and children with imprinting disturbances [63].

### 6.3. Environmental Risk Factors for ImpDis

In addition to genetic factors, the impact of the environment on imprinting signatures becomes increasingly apparent. However, the identification of environmental factors causing altered imprinting patterns is challenging, though strong evidence of the role of nutritional status and chemical pollutants on pregnancy has been published (for review, see [64]. In particular, with assisted reproduction technologies (ARTs), an increased probability of imprinting disturbances has been identified, at least for those associated with the 11p15.5 DMRs [65]. The causal link between ARTs and ImpDefs is currently unknown, but given the procedure of ARTs coincides with key steps in the establishment and maintenance of imprinting marks, this is conceivable.

## 7. Diagnostic Testing for ImpDis in a Reproductive Context

Molecular diagnostic testing for ImpDis is commonly offered to confirm a clinical diagnosis and to identify carriers of pathogenic variants which cause predisposition to an increased probability of having a child with an ImpDis. Furthermore, ImpDis testing is discussed in a prenatal context, either because of fetal and maternal health issues during pregnancy or because of a family history of ImpDis [18].

The diagnostic testing of ImpDis is challenging due to the clinical and molecular overlap between disorders and their heterogeneous molecular basis, which makes the decision on the appropriate tests difficult [66].

Furthermore, cellular mosaicism impedes the detection of ImpDefs and upd(11)pat and might result in false negative testing results (e.g., in BWS; see [67]). In addition to the insufficient sensitivity of some testing methods, the time of mosaic formation and severe differences in mosaic distribution between different tissues are the reasons for false negative testing results. Thus, a negative testing result never discounts the possibility of an ImpDef or a upd(11)pat (for review, see [68]).

Several scenarios have been suggested as reasons for invasive prenatal ImpDis testing [18]. They comprise ultrasound findings associated with ImpDis (growth abnormalities, macroglossia, abdominal wall effects; in context with ART), a family history of an ImpDis due to an inherited molecular alteration (SNV, SV, recurrent children with ImpDis, recurrent miscarriages/hydatidiform moles) and specific chromosomal findings affecting imprinted regions (e.g., familial SV, confined placental trisomy mosaicism).

Due to these aspects and the complexity of imprinting disturbances and their underlying mechanisms, prenatal genetic testing for ImpDis has to be accompanied by genetic counselling. This counselling has to consider the reason for referral and the technical limitations of prenatal molecular analysis. Furthermore, the predictive value of such a test for the clinical outcome, as well as its consequence for the management of the pregnancy, has to be discussed prior to the test. In prenatal management, fetal imaging should be the major diagnostic tool on which decisions on the management of the pregnancy should be based.

Genetic testing for maternal effect variants is a specific situation. In fact, the pathogenic potential of SCMC variants is meanwhile accepted for families with a massive history of recurrent miscarriages, children with ImpDefs and/or aneuploidies carrying maternal effect variants [69]. However, prediction of the recurrence risk for the aforementioned reproductive issues is not possible, and oocyte donation has been suggested as a therapeutic option [70]. Nevertheless, the option of genetic testing for MLID and the consideration of oocyte donation currently account only for a small number of families, and each family and situation requires a careful anamnesis of the relevant reproductive and family history.

## 8. Conclusions

Due to the central role of genomic imprinting in human pregnancy and embryogenesis, its disturbance not only affects the carrier of the alteration but also the course of pregnancy and even the health of the mother. Due to the complexity of imprinting regulation, each pregnancy or patient with suspected altered genomic imprinting requires a specific workup to identify the precise molecular cause and also careful clinical documentation.

Additionally, the underlying molecular genetic and epigenetic variants can be inherited, but prediction of the exact pregnancy complications is difficult, as they can cause miscarriages, aneuploidies, health issues for the mother and ImpDis in the child.

With the use of high-throughput technologies in the research on and genetic diagnostics of imprinting disorders, the spectrum of molecular alterations will be ascertained, as well as their underlying molecular causes and physiological consequences. Altogether, this progress will allow more personalized clinical management for patients but also targeted genetic and reproductive counselling of families.

## Figures and Tables

**Table 1:** Overview of established and assumed imprinting disorders for which prenatal features and complications have been reported. (^a^ These genes on chromosome 7 are targeted using routine diagnostic testing, but their role in the etiology of SRS is currently unclear. ^b^ The MEG3/DLK1:IG-DMR is the more relevant DMR in 14q32, but is not routinely targeted due to its molecular complexity. ^c^ This DMR has been suggested to be relevant to aupd(16)mat phenotype. ^d^ Reports on placental morphology for upd(16)mat are not available, but due to the origin of upd(16)mat from trisomy 16 rescue and associated placental trisomy 16 (mosaicism), a placental phenotype might be delineated. NR, not yet reported; ND, not determined; upd(6)pat, paternal uniparental disomy of chromosome 6 (abbrevations are similar for other upds); DMR, differentially methylated region; LOM, loss of methylation; GOM, gain of methylation; LOI, loss of imprinting; GoF, gain of function; LoF, loss of function; SNVs, single nucleotide variant; CNV, copy number variant).

Imprinting Disorder OMIM	Chromosome	ImpDis-Specific DMR	Intrauterine Phenotype	Preterm Delivery	Placental Phenotype	Molecular Defects	Frequencies among Molecularly Confirmed Cases
Transient neonatal diabetes mellitus (TNDM) 601410	6q24	*PLAGL1*:alt-TSS-DMR	Growth restriction, abdominal wall defects	30% < 37 gw [6]		upd(6)pat	41%
						dup(6q24)pat	29%
						*PLAGL1*:alt-TSS-DMR: LOM	30%
Silver–Russell syndrome (SRS) 180860	7	*GRB10*:alt-TSS-DMR ^a^, *MEST*:alt-TSS-DMR ^a^	Growth restriction	Yes [7]	Calcification [8]	upd(7)mat	15.8%
	11p15.15	*H19/IGF2*:IG-DMR			Mice: placental undergrowth, defective vascularization [9]	upd(11p15)mat	single cases
						del(11p15)pat	single cases
						dup(11p15)mat	2.3%
						*H19/IGF2*:IG:DMR: LOM	67.6%
						*CDKN1C* (GoF), *IGF2*, *HMGA2*, *PLAG1:* SNVs, CNVs	several cases
Beckwith–Wiedemann syndrome (BWS) 130650	11p15.5	*H19/IGF2*:IG-DMR	Overgrowth, polyhydramnion, abdominal wall defects, placental mesenchymal dysplasia, (mother: preeclampsia)	Yes [10]	Placental mesenchymal dysplasia; placentomegaly	upd(11p15)pat	19.5%
		*KCNQ1OT1*:TSS-DMR				dup(11p15)pat	<1%
						*H19*/IGF2:IG-DMR: GOM	11.8%
						*KCNQ1OT1*:TSS-DMR: LOM	64.0%
						*CDKN1C*: (LoF) SNVs: sporadic, familial	5%, 40%
Temple syndrome (TS14) 616,222	14q32	*MEG3/DLK1*:IG-DMR	Growth restriction	30% [11]		upd(14)mat	54.0%
		*MEG3*:TSS-DMR				del(14q32)pat	12.2%
						*MEG3/DLK1*:IG-DMR: LOM	33.8%
Kagami–Ogata syndrome (KOS14) 608149	14q32	*MEG3/DLK1:IG-DMR* ^b^	Overgrowth, polyhydramnion, abdominal wall defects, placentomegaly	Yes [11]	Placentomegaly	upd(14)pat	51.5%
		*MEG3:TSS-DMR* ^b^				del(14q32)mat	21.9%
						*MEG3/DLK1*:IG-DMR: GOM	26.6%
Prader–Willi syndrome (PWS) 176270	15q11q13	*SNRPN*:alt-TSS-DMR	SGA: 53% [12]	26% [13]		del(15q11q13)pat	70–75%
						upd(15)mat	25–30%
						*SNURF*:TSS-DMR: GOM	1%
upd(16)mat	16	*ZNF597*:TSS-DMR ^c^	Growth restriction (mother: preeclampsia [14])	Small placenta? ^d^ [15]		upd(16)mat	100%
Pseudohypoparathyroidism:	20q13	*GNAS-NESP*:TSS-DMR	Growth restriction	NR			
PHP1B (iPPSD3) 603233						upd(20q13)pat	2.7%
		*GNAS-AS1*:TSS-DMR				broad LOI (all GNAS DMRs)	38%
		*GNAS-XL*:Ex1-DMR				broad LOI (all GNAS DMRs)	rare
		*GNAS A/B*:TSS-DMR				*GNAS A/B*:TSS-DMR: LOM	13.5%
PHP1A/PPHP/POH (iPPSD2) 103580/612463						*GNAS:* LoF SNVs and CNVs	37.7%
Mulchandani–Bhoj–Conlin syndrome (MBCS) 617352	20	?	Growth restriction [16]	NR		upd(20)mat	ND

**Table 2:** Examples of imprinted genes in mice and humans and their involvement in prenatal growth and metabolism (leaned on [49]). (^a^ Expression in pregnancy; later in life, the imprinting status might be different. ^b^ Disturbances of DMRs in these genes are associated with human ImpDis. ^c^ These genes have been suggested as candidates for SRS, but their functional impact has not yet been proven. ^d^ It should be noted that *PEG3* itself is commonly not altered in TNDM, but that TNDM patients carrying *ZFP57* variants exhibit a specific episignature, which includes *PEG3* LOM).

Mouse		Human				Findings in Mice: Function in	
Gene	Expressed Parental Allele ^a^	Gene	Expressed Parental Allele ^a^	Chromosome	ImpDis ^b^	Placenta	Embryo
*Zac1*	paternal	*ZAC1*	paternal	6q24	TNMD	Promotes growth	Promotes growth
*Grb10*	maternal	*GRB10*	maternal	7p13	SRS ^c^	Suppresses growth	Suppresses growth
*Peg10*	paternal	*PEG10*	paternal	7q21		Structural organization	
*Mest/Peg1*	paternal	*MEST*	paternal	7q32	SRS ^c^	Suppresses growth	Promotes growth
*Igf2*	paternal	*IGF2*	paternal	11p15.5	BWS/SRS	IGF2-P0-specific transcript: promotes growth	Promotes growth
*Cdkdn1C*	maternal	*CDKN1C*	maternal	11p15.5	BWS/SRS	Suppresses growth	No effect
*Kcnq1ot1*	Paternal	*KCNQ1OT1*	paternal	11p15.5	BWS	Cis silencing of neighbouring imprinted genes	
*Mash2*	maternal	*ASCL2*	maternal	11p15.5		Structural organization	
*Tssc3*	maternal	*PHDLA2*	maternal	11p15.5		Promotes growth	
*Gtl2*	maternal	*MEG3*	maternal	14q32	TS14/KOS14		Promotes growth
*Dlk1*	paternal	*DLK1*	paternal	14q32.2			Promotes growth
*Peg3*	paternal	*PEG3*	paternal	19q13.43	TNDM ^d^	Suppresses growth	Promotes growth

## Data Availability

Not applicable.
